# The effect of a seven-step rehabilitation training program on cardiac function and quality of life after percutaneous coronary intervention for acute myocardial infarction

**DOI:** 10.12669/pjms.38.1.4945

**Published:** 2022

**Authors:** Xuemei Peng, Jianhui Zhang, Lihong Wan, Hui Wang, Wuning Zhang

**Affiliations:** 1 Xuemei Peng, Department of Cardiology, The First Hospital of Fangshan District, Beijing, Beijing 102400, P.R. China; 2 Jianhui Zhang, Department of Cardiology, The First Hospital of Fangshan District, Beijing, Beijing 102400, P.R. China; 3 Lihong Wan, Department of Cardiology, The First Hospital of Fangshan District, Beijing, Beijing 102400, P.R. China; 4 Hui Wang, Department of Cardiology, The First Hospital of Fangshan District, Beijing, Beijing 102400, P.R. China; 5 Wuning Zhang, Department of Cardiology, The First Hospital of Fangshan District, Beijing, Beijing 102400, P.R. China

**Keywords:** Acute myocardial infarction, After percutaneous coronary intervention, Living ability, Seven-step rehabilitation training

## Abstract

**Objectives:**

To study the effect of a seven-step rehabilitation training program on cardiac function and quality of life in acute myocardial infarction (AMI) patients after percutaneous coronary intervention (PCI).

**Methods:**

In this study one hundred AMI patients undergoing emergency PCI at The First Hospital of Fangshan District between June 2019 and June 2020 were included. Patients were retrospectively divided into two equal groups based on the type of physiotherapy regiment. The training group included patients who underwent seven-step rehabilitation training while the control group had patients who received routine nursing. Left ventricular ejection fraction (LVEF), self-care capability, hospitalization duration, quality of life, and adverse cardiac event incidence were compared.

**Results:**

The number of patients with LVEF values ≥ 50% was significantly higher in the training group after one week of training. Training group patients also showed decreased hospitalization duration and larger improvement in self-care capacity scores. At three months after training, training group patients had overall superior quality of life and lower incidence rates of arrhythmia and angina pectoris.

**Conclusion:**

The seven-step rehabilitation training program has a significant effect on improving AMI patient quality of life and cardiac function post-PCI, and is worthy of continued study and promotion.

## INTRODUCTION

Acute myocardial infarction (AMI) is caused by myocardial necrosis resulting from the interruption—often by coronary atherosclerotic plaques-of continuous myocardial blood supply. Percutaneous coronary intervention (PCI) is widely used clinically as an effective method to quickly restore coronary blood supply. However, while studies^1^,^2^ have noted that rehabilitation should be actively employed post-PCI, early postoperative rehabilitation exercise has significant clinical effects on acute cardiac infarction.^1^ Interestingly, a seven-step rehabilitation program applied for cardiac rehabilitation has been previously shown to improve patient self-care capability and cardiac function.^3^ This retrospective study examined the influence of the seven-step rehabilitation program versus conventional rehabilitation on patient short- and long-term outcomes following AMI and subsequent PCI intervention.

## METHODS

One hundred patients with AMI that were treated at The First Hospital of Fangshan District with PCI via the radial artery between June 2019 and June 2020 were included in the study. Medical records of the patients were reviewed and the patients were divided into two equal groups (50 patients per group) based on the rehabilitation regiment. A training group received seven-step rehabilitation training, and the control group underwent conventional rehabilitation. The control group contained 39 males and 11 females and averaged 54.2 ± 8.4 years of age. Of these 50 individuals, 28 received one stent, 18 received two, and four received three. Twenty-six of these individuals smoked and 20 consumed alcohols. The training group contained 41 men and 9 women, averaging 56.1 ± 7.5 years of age. Of these 50 individuals, 26 received one stent, 19 received two stents, and five received three stents. Twenty-four of these individuals smoked and 22 consumed alcohols. The study was approved by the Ethics Committee of The First Hospital of Fangshan District.

### Inclusion criteria:

1. Complete medical history
2. Patient treatment conformed to the Guidelines for Diagnosis and Treatment of Acute Myocardial Infarction formulated by the Chinese Medical Association
3. Grade I and II cardiac function, based on the New York Heart Association classification*:* Grade I: patients with cardiac disease but without resulting limitations of physical activity. Grade II: Patients with cardiac disease resulting in slight limitation of physical activity^4^
4. No chest pain, arrhythmia, or electrocardiogram changes in the eight hours prior to training
5. No decompensation indicative of heart failure;
6. Myocardial injury marker levels did not further increase after intervention.

### Exclusion criteria:

1. Patients had congenital heart disease
2. Chronic obstructive pulmonary disease and other serious lung diseases
3. Hepatic and renal insufficiency
4. Malignant tumor patients
5. Mental illness, consciousness disorder, or inability to cooperate

### Nursing intervention methods:

#### Control group:

Control group patients received routine nursing consisting of ECG monitoring for myocardial ischemia and arrhythmia over the first 24 hours, routine administration of antiplatelet drugs, blockers, lipid-regulating drugs, and other medications, application of compression bandaging with an elastic bandage, and encouraging patients to manage their diet and hydration habits with the aim of keeping stools unobstructed.

#### Training group:

The training group received a seven-step rehabilitation training method, following hospital guidelines. Detailed progress report was available for each patient, included in the study. Prior to initiation, patients heart rate was confirmed by ECG to be under 100 beats/minutes, with normal blood pressure and no arrhythmia. Step 1: on the first day of training, patients carried out active and passive exercise of limbs on the bed. Patients were allowed to wash hands, wash face, eat, and use bedpan while in bed. Patients were also allowed to sit up after raising the head of the bed or on their own power for 15-30 min up to twice a day with the help of medical workers. Step 2: on the second day of training, in addition to the previous activities, patients were allowed to wash and wipe themselves by the bed, read for under 15 minutes, and sit up for 15-30 minutes, twice a day. Step 3: on the third day of training, in addition to the previous activities, patients were allowed to walk slowly for 30 meters and try to walk to the toilet. Step 4: on the 4th day of training, in addition to the previous activities, patients walked in place 10-15 times, went to the bathroom by themselves, and tried to wash his body with warm water. Step 5: on the 5th day of training, in addition to the previous activities, patients were allowed to walk 150 meters and tried to take three steps on the training escalator, twice a day, as well as go to the bathroom for cleaning. Step 6: on the 6th day of training, in addition to the previous activities, patients were allowed to walk 150 meters and tried to take five steps on the training escalator, twice a day. Step 7: on the 7th day of training, in addition to the previous activities, patients were allowed to walk 150 meters and tried to take 10 steps on the training escalator, twice a day.

The day’s training course was stopped immediately if patients felt palpitations, chest tightness, shortness of breath, dizziness, or other symptoms during training. Patients were not permitted to advance to the next step if any of the following signs were apparent after training: heart rate (as monitored via ECG) ≥ 110 beats/min, systolic blood pressure fluctuations exceeding 20 mmHg from resting values, ST segment elevation) ≥ 0. 2mV or depression) ≥ 0. 1 mV. In these situations, the training plan successfully completed during the prior day would be implemented.

### Observation indicators:

All patients in the study were diagnosed with ST Elevation Myocardial Infarction (STEMI). The baseline values of cTnT and cTnI were (1.24 ± 0.13) μ g/L and (25.68±1.56) μ g/L, respectively.

### Left ventricular ejection fraction:

Records of baseline and post-training left ventricular ejection fraction (LVEF) measurements obtained using echocardiography were analyzed for each patient. An LVEF under 50% indicated decreased ventricular function.

### Immediate daily quality of life:

The activity of daily living (ADL) scale was used to assess quality of life before and after training. ADL evaluates a total of 10 activities (defecation, urination, eating, toilet use, bathing, dressing, decoration, transfer, activity, and climbing stairs), with each item scored between 0-10. A maximum score is 100 points, with a score over 60 indicating self-sufficiency. Scores between 41-60 indicate some assistance is required; 21-40 indicates considerable assistance required; and scores under 20 indicate complete dependency. ADL scores of two groups were retrospectively compared.

### Long-term quality of life and adverse cardiac event incidence:

A single follow-up was conducted three months post-discharge. Outpatient nurses conducted one-on-one interviews touching on the areas of environment, physiology, independence, and psychology, using the answers to fill in a standard summary form. Doctors confirmed the presence or absence of angina pectoris, heart failure, arrhythmia, or recurrent coronary stenosis .

### Statistical Analysis:

All data were analyzed using SPSS 19.0 statistical software. The counting data is expressed in the form of rate (n%), *χ^2^* is the test comparison result, x±s is the mean and standard deviation of measurement data, t is the comparison result of group test. When p < 0.05, the difference was statistically significant.

## RESULTS

No statistical significance in age, gender, number of stents implanted, or history of tobacco/alcohol usage was found between the two groups (Table-I).

**Table I:** Patient characteristics.

Project	Control group (n=50)	Training group (n=50)	2/t	P
Male	39 (78.0)	41 (82.0)	2=0. 250	0.617
Female	11 (22.0)	9 (18.0)	2=0. 250	0.617
Age (years)	54.2±8. 4	56.1±7. 5	T=1. 193	0.236
One stent	28 (56.0)	26 (52.0)	2=0. 160	0.688
Two stents	18 (36.0)	19 (38.0)	2=0. 040	0.836
Three stents	4 (8.0)	5 (10.0)	2=0. 120	0.727
Smoking (%)	26 (52.0)	24 (48.0)	2=0. 160	0.689
Drinking alcohol (%)	20 (40.0)	22 (44.0)	2=0. 160	0.685

### LVEF:

There was no difference in baseline LVEF between the two groups prior to training. After training, the number of patients with LVEF values ≥ 50% in the training group was significantly higher than in the control group (Table-II).

**Table 2:** Comparison of left ventricular ejection fraction (%) between the two groups (n %).

Group	Number of cases	Before training		After training		2	P

		< 50%	≥ 50%	< 50%	≥ 50%		
Training group	50	26 (52)	24 (48)	14 (28)	36 (72)	6.000	0.014
Control group	50	25 (50)	25 (50)	24 (48)	26 (52)	0.040	0.841
2	-	0.040 0.841		4.240 0.039		-	-
P	-			- -			

Training group patients were hospitalized for significantly shorter durations than control group patients. ADL scores increased in both groups after training, but the increase was greater in the training group (Table-III)***-*** At three months post-training, quality of life for training group patients was better than control group patients (Table-IV).

**Table III:** Hospitalization time and ADL scores for patients receiving conventional rehabilitation and seven-step rehabilitation.

Group	n	Hospitalization time (days)	ADL (points)	Before and after training	T	P
Training group	50	7.22 ± 0. 85	55.34 ± 10. 24	91.77 ± 8. 34	19.505	0.000
Control group	50	9.53 ± 1. 23	55.19 ± 9. 02	87.09 ± 11. 74	15.236	0.000
T	-	10.925	0.078	2.298	-	-
P	-	0.000	0.938	0.024	-	-

**Table IV:** Comparison of quality-of-life summary scores for the two groups at three months post-training.

Group	n	Environmental	Physiological	Independence	Psychological	Total Score
Training group	50	24.8 ± 2. 33	26.3 ± 0. 75	14.7 ± 2. 61	22.5 ± 1. 13	85.3 ± 4. 21
Control group	50	20.9 ± 3. 24	23.5 ± 0. 91	9.5 ± 2. 83	18.2 ± 1. 19	69.1 ± 5. 65
T	-	6.910	16.790	9.551	18.528	16.258
P	-	0.000	0.000	0.000	0.000	0.000

Occurrence of arrhythmia and angina pectoris in the training group was lower than that in the control group at three months after training. No difference was noted in the rates of heart failure and recurrent coronary stenosis (Table-V).

**Table V:** Adverse events in AMI patients at three months post-training.

Group	n	Angina pectoris	Heart failure	Arrhythmia	Recurrent coronary stenosis
Training group	50	4 (8.0)	2 (4.0)	3 (6.0)	2 (4.0)
Control group	50	14 (28.0)	7 (14.0)	11 (22.0)	4 (8.0)
2	-	6.780	3.050	5.320	0.710
P	-	0.009	0.081	0.021	0.400

## DISCUSSION

China is facing a growing aging population, leading to increased incidence and mortality from cardiovascular and cerebrovascular diseases, including AMI.^5^,^6^ PCI via the radial artery can quickly restore coronary blood supply and improve myocardial blood supply, and is therefore recommended in AMI treatment guidelines. PCI is conducive to early rehabilitation exercise, and some studies have shown that early rehabilitation is beneficial for AMI patients.^1^,^7^,^8^

This retrospective study compared the effectiveness of the seven-step rehabilitation training proposed by the American Heart Association (AHA) with routine nursing after PCI in AMI patients. It found that after one week of rehabilitation training, the number of patients with LVEF ≥ 50% was significantly higher in patients receiving seven-step rehabilitation training. The early implementation of gradual exercise may enable patients to quickly recover physical fitness and promote cardiovascular regulation under relatively safe conditions. It may also promote the formation of collateral circulation and increase ventricular systolic function, thereby increasing LVEF.^9^ In the past, many researches adopted cardiac rehabilitation nursing to improve ventricular function, and seven step rehabilitation training was integrated into daily activities in a step-by-step way, which not only ensured the rehabilitation needs of patients, but also changed the absolute bedridden lifestyle of patients.^10^ It is suggested that in the nursing work, it is necessary to cultivate patients’ self-care habits, improve patients’ self-awareness, and lay the foundation for long-term nursing after discharge, which is relatively lacking in the current domestic nursing work. Therefore, combined with foreign nursing experience, we can promote long-term care of patients after operation and discharge, and provide clinical nursing quality.

This study also found that implementation of the seven-step rehabilitation training program significantly reduced hospital stay durations. In alignment with this, the ability of patients to take care of themselves also improved. It is possible that the incremental elevation of physical activity bears similarities to daily living activities and is conducive to developing self-care capability.^1^,^11^ In recent years, with the progress of nursing after PCI, effective nursing methods have better improvement effect on adverse cardiac events, and postoperative adverse cardiac events are the risk factors affecting the prognosis of patients with AMI. Therefore, the attention to postoperative nursing can help to improve the clinical prognosis to a certain extent, and realize the professionalism and value of nursing, which is of great significance to the clinical nursing function.

These trends persisted at three months following intervention, indicating that early rehabilitation exercise can significantly improve patient self-care capabilities over a prolonged duration, possibly due to promoting the recovery of motor neurons.^12^ Furthermore, it is possible that improved cardiac function observed after seven-step rehabilitation training may be related to the observed reduction in arrhythmia and angina pectoris incidence.

### Limitations of the study:

It is the small sample size of this study from a single center. Although it has certain clinical significance, it needs to further expand the sample validation. In addition, the way of rehabilitation training has more reference, but there is no unified standard. The bias of nursing methods caused by individual factors of patients cannot be avoided. Therefore, the clinical application of its nursing method needs to be further improved.^13^

## CONCLUSION

The seven-step rehabilitation training regimen can significantly improve cardiac function in AMI patients post-PCI, thereby promoting improved quality of life and reduction of adverse cardiac events. However, additional studies are needed to strengthen the findings of this study, given its small sample size.

### Author’s Contributions:

**XP and JZ:** Conceived and designed the study and is responsible for integrity of the study.

**LW, HW & WZ:** Collected the data and performed the analysis.

**XP and JZ:** Was involved in the writing of the manuscript.

All authors have read and approved the final manuscript.
